# Surface modification on MoO_2+*x*_/Mo(110) induced by a local electric potential

**DOI:** 10.1038/s41598-019-42536-9

**Published:** 2019-04-17

**Authors:** Sergey I. Bozhko, Killian Walshe, Natalia Tulina, Brian Walls, Olaf Lübben, Barry E. Murphy, Vladimir Bozhko, Igor V. Shvets

**Affiliations:** 1 Institute of Solid State Physics, Russian Academy of Sciences, Chernogolovka, Moscow District 142432 Russia; 2 School of Physics and Centre for Research on Adaptive Nanostructures and Nanodevices (CRANN), Trinity College Dublin, Dublin 2, Ireland

**Keywords:** Electronic properties and materials, Surfaces, interfaces and thin films, Atomic and molecular physics

## Abstract

Oxygen adatoms on the MoO_2+*x*_/Mo(110) surface are observed to be removed when a sufficiently large bias is applied between the scanning tunneling microscope tip and the surface. Experimental observations, such as the bias polarity dependence of adatom removal and the observation of an intermediate state, indicate that the adatom penetrates the surface oxide layer. Through the comparison of finite element method simulations with various experimental relationships, the electric field is concluded to be the sole contributor to adatom penetration into the surface oxide layer. The energetic barrier to this process is estimated to be approximately 0.45 eV in magnitude. Furthermore, the resolution of this phenomenon is on the atomic scale: individual adatoms can undergo surface penetration whilst their nearest neighbour adatoms, separated by 5 Å, are unaffected. The mechanism reported here has the advantages of not strongly influencing the substrate and is exceptionally localised, which can be beneficial for the synthesis of single atom devices.

## Introduction

Molybdenum is a crucial element in many high-performance alloys and steels^1,2^. Its compounds are utilized in a wide variety of applications such as fertilisers^3^, nanowires^4^ and catalysts^5^. Molybdenum oxides are important catalysts, catalytic applications include hydrotreating of oil to reduce the sulphur, nitrogen and aromatics content, yielding cleaner more efficient fuels^6^. MoO_2_ is a conductor which has been reported to act as a catalyst in several different reactions including the dehydrogenation of alcohols^7^, the reformation of hydrocarbons^8,9^ such as biodiesel^10^ and is used for the partial oxidation of aviation fuel^11^. The catalytic activity shown of MoO_2_ is due to the transfer of nucleophilic oxygen ions from the oxygen sub-lattice to the catalytically active surface where they are then consumed to sustain the redox cycles^12^. The oxygen content is an important factor in catalytic activity. For example, the catalytic activity of MoO_*x*_ in converting methanol to formaldehyde is determined by its oxygen coordination^13^: MoO_2_ is a poor catalyst in comparison to MoO_3_. The presence of Mo^6+^ is the critical factor in obtaining a high formaldehyde yield rate. The ability to control which MoO_*x*_ species is present on the surface has important applications for oxidation and reduction reactions. MoO_2_ can also be used as a nanostructure template^14^.

Individual atom surface modification using the Scanning Tunneling Microscopy (STM) tip was first famously demonstrated by ‘writing’ “IBM” with Xe atoms on a Ni(110) surface^15^. Adsorbates on metal and semiconductor surfaces can be manipulated using the electric field generated between the STM tip and the surface^16^, tunneling electrons^17^ from the tip to the surface or a combination of both. Electric field induced diffusion^18,19^, desorption^20^ and switching^21,22^ of atoms and molecules on metal and semiconductor surfaces have been previously observed using STM techniques, however, adatom penetration into the surface has yet to be realised. The vertical movement of an atom through a surface has important applications in the field of quantum computing: in order to manipulate and read out a quantum state, such as the nuclear spin of an atom, requires the precise placement of atoms within a lattice^23^. Current methods involve creating a high precision hydrogen mask on a Si surface using the STM tip and then dosing the system with PH_3_ and subsequently growing Si over-layers^24^. Other methods involve firing high energy (14 keV) P ions at a Si(100) substrate to implant them within the Si lattice^25,26^. Atom manipulation with the STM tip offers the high resolution required to precisely place an atom within a lattice, provided the tip can impart a sufficient force to result in the atom penetrating the surface when a voltage pulse is applied.

In a previous publication, we have demonstrated the ability to ‘write’ on the MoO_2+*x*_/Mo(110) surface. In that work, oxygen adatoms were removed from the surface via pulsing with the STM tip^27^. Pulsing was carried out in the tunneling regime, where both tunneling electrons and the electric field between the tip and surface could contribute to adatom removal. In that regime, the oxygen adatoms were concluded to be removed from the surface via inelastic electron tunneling. However, we will demonstrate that is also possible to observe the removal of the oxygen adatoms when not in the tunneling regime. In the current work, we present results based on pulsing adatoms on the MoO_2+*x*_/Mo(110) surface outside of the tunneling regime, where the electric field is the only candidate mechanism for adatom removal. There are two possibilities for the observed removal of oxygen adatoms from the surface, either the adatom escapes from the surface to the vacuum via desorption or the adatom penetrates the surface. The desorption of atoms from a surface to a vacuum using an electric field is demonstrated in Field Ion Microscopy (FIM). Protruding atoms evaporate from the microscope tip in a process called field evaporation. Field evaporation can be employed to examine the binding energy of surface atoms^28^, typically in the range 2–5 V/Å^29^. Metals and their compounds can have a potential barrier to the incorporation of adsorbates such as oxygen into and beneath the surface layer. The magnitude of this barrier affects surface phenomena such as catalysis, corrosion, friction, the kinematics of oxidation and the transition from physisorption to oxidation states. If a barrier to oxygen penetration is present Cabrera-Mott theory describes the growth of a thin film oxide layer^30^. The transport of oxygen ions through an oxide film has applications in the field of memristors^31,32^. These circuit elements have the unique ability to vary their resistance based on the applied current.

The purpose of this study is to understand the mechanism behind the removal of adatoms from the MoO_2+*x*_ surface outside of the tunneling regime and to understand why the structure of the oxide layer allows for this process to occur. We have studied the effect of applying an electric field to an oxygen adatom on the MoO_2+*x*_/Mo(110) surface with no influence from the tunneling current and we conclude that it is possible to induce the transition of an oxygen ion from the surface to an underlying oxide layer. Finite element method (FEM) simulations are employed to show that an electric field alone can replicate the experimental results. Inducing adatom penetration through a surface by means of an applied local electric field is a new mechanism to the best of our knowledge and this method has potential applications in the fabrication of single-spin devices such as qubits for quantum computing as well as in the field of single molecule engineering^33,34^, which strives to assemble atomic-scale structures from their individual atomic components using the STM tip.

## Results

### Experimental Results

#### Adatom Removal

**Figure 1:**
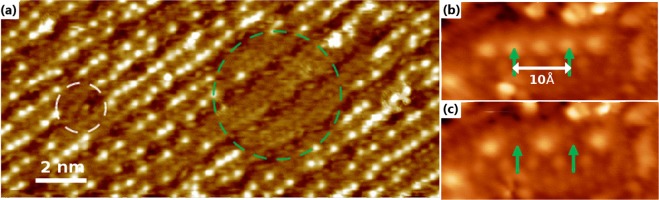
(**a**) Oxygen adatom removal via pulsing at *V* = 3.5 V (white circle) and *V* = 4.5 V (green circle). **(b)** STM image of a row of oxygen adatoms on the MoO_2+*x*_ surface, before single pulses are applied to each atom highlighted in green. **(c)** Individual adatoms are removed from the surface following pulsing, while their neighbours remain in place. The peak to peak distance between adatom features is 5 Å, demonstrating the high resolution of this method.

Annealing a single crystalline Mo(110) sample in an oxygen partial pressure results in the formation of a thin film of MoO_2_ forming on the surface. Oxidising the MoO_2_/Mo(110) sample at room temperature results in the formation of an oxygen-rich MoO_2+*x*_/Mo(110) surface. The additional oxygen takes the form of adatoms on top of the O-Mo-O trilayer structure. Applying a single voltage pulse (positive sample bias) with the STM tip when outside of the tunneling regime can lead to a number of oxygen adatoms being removed from the surface at once, as seen in Fig. 1(a). In this image we do not observe an increase in the density of oxygen adatoms in the vicinity of the pulsed area, indicating that there is no lateral diffusion. This can also be viewed in a previous work^27^ where larger scale images are presented. No tunneling current is present during a bias pulse due to applying a sufficiently large vertical offset between the tip and surface, further details relating to the pulsing conditions and the sample preparation can be found in the methods section. The diameter of the circular area from which oxygen adatoms are removed depends on the magnitude of the pulse voltage. Individual adatoms can also be removed even when neighbour adatoms were separated by just 5 Å. The high resolution of oxygen adatom removal is depicted in Fig. 1(b,c). A single pulse is applied to each adatom highlighted in Fig. 1(b) resulting in their removal, seen in Fig. 1(c). Oxygen adatom removal was only observed at positive sample bias. Given that the oxygen adatom is negatively charged, the applied electric field imparts a force on the adatom acting towards the bulk.

#### Intermediate State

**Figure 2:**
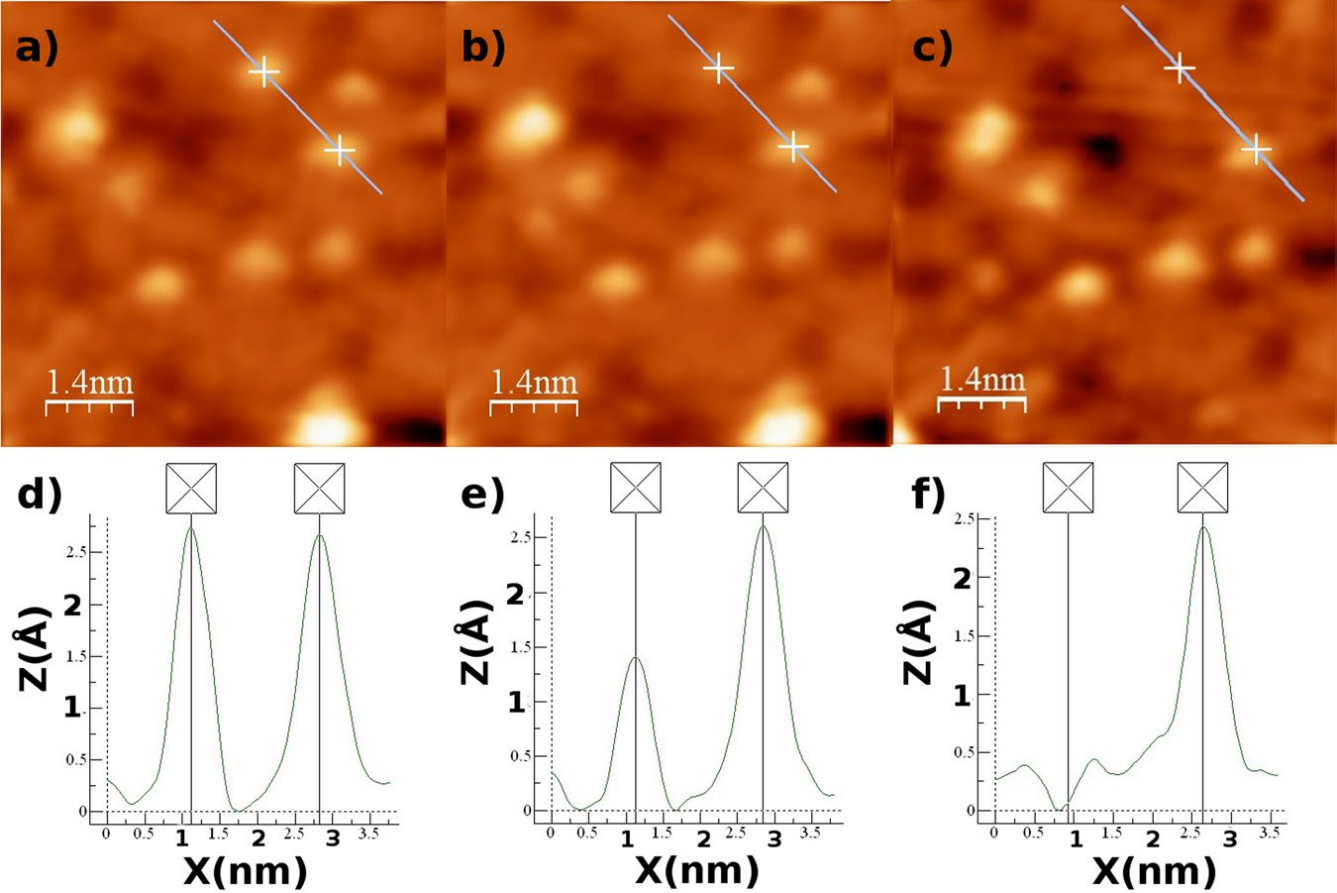
STM images of the MoO_2+*x*_/Mo(110) oxide surface. **(a**,**d)** correspond to the STM image and line scan respectively before a pulse is applied to the adatom positioned at the cross at the top of the STM image presented in **(a)**, its relative height decreases as seen in **(b**,**e)**. Another pulse is then applied to the same adatom. Finally in **(c**,**f)** it is clear that the aforementioned adatom has been removed from the surface.

After pulsing is performed the oxygen adatom is either removed from the surface or still in its original location on the surface, depending on the magnitude of the bias pulse. However, on occasion pulsing has been observed to change the relative height of the oxygen adatom as observed by STM. In Fig. 2, three STM images and three lines profiles are presented. From left to right the STM images correspond to before pulsing (Fig. 2(a)), after one pulse (Fig. 2(b)) and after a second pulse is applied in the identical position (Fig. 2(c)). The sequence of line profiles (Fig. 2(d–f)) illustrate that the adatom is not fully removed from the surface after the initial pulse, but sits in an intermediate state (Fig. 2(e)). This intermediate state is suspected to be a different oxide compound that lies within the topmost molybdenum layer. After a second pulse is applied (Fig. 2(f)) the adatom is fully removed from the surface. This intermediate state has only been observed on a few occasions, when the pulsing bias is close to the threshold voltage, in contrast to the oxygen removal phenomenon. The change in relative height of the feature may be due to a change in the adatom position and/or a change in the electronic state of the adatom, through ionisation or a chemical reaction resulting in an oxide compound being formed.

#### Spot Size vs. Applied Bias

**Figure 3:**
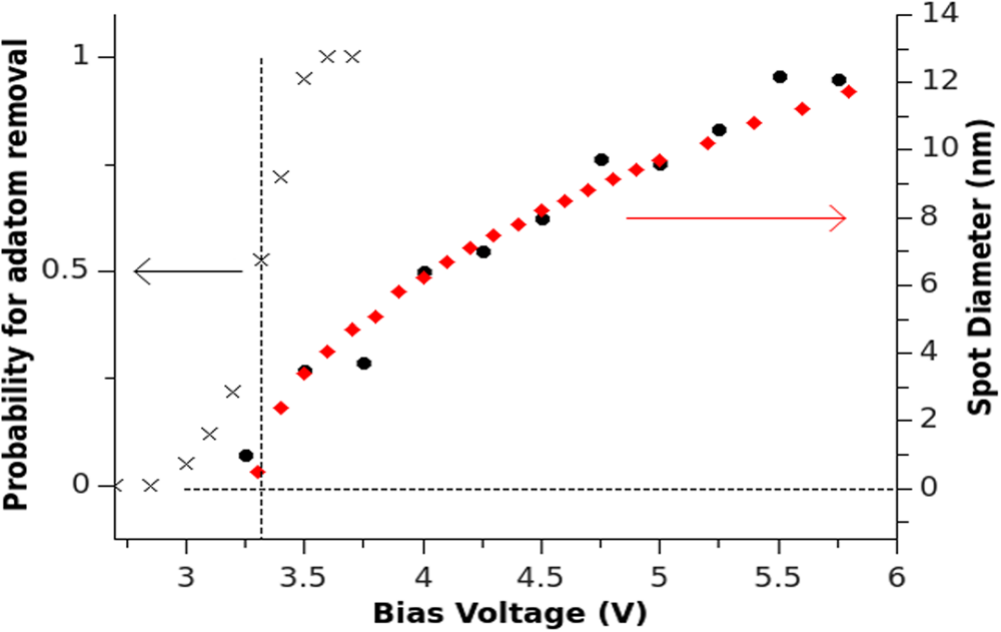
Grey crosses: The probability to remove an adatom increases sharply at the threshold voltage. Vertical dashed line: The threshold voltage corresponds to removing an adatom with a probability of 0.5 or the minimum spot size of 0.5 nm. Black circles: Experimentally, the spot diameter increases as the applied bias voltage is increased. Red diamonds: FEM simulations of the relationship between the spot diameter and applied bias voltage are in agreement with the experimental result.

The minimum voltage required to remove an adatom is referred to as the threshold voltage, which depends on conditions such as the tip-surface separation. The threshold voltage for the removal of oxygen adatoms can be measured in two ways. Firstly, by systematically increasing the bias voltage and monitoring the increase in the spot diameter, a non-linear dependence is obtained as indicated by the black circles in Fig. 3. The threshold voltage is estimated by extrapolating the bias voltage from this relationship to a minimum spot size of 0.5 nm, which corresponds to a single oxygen adatom atom being removed. This method gives a qualitative estimate of the threshold voltage. Secondly, the threshold voltage can also be estimated by measuring the pulse voltage at which the probability of removing an adatom is roughly 50%. 30 individual pulsing experiments were performed at different points on the surface. For each experiment, the pulse voltage was increased until adatom removal was achieved. The grey crosses in Fig. 3 depict the probability of adatom removal as a function of the pulse voltage. The probability that an oxygen adatom will be removed by applying a pulse sharply increases at the threshold voltage. Both methods indicate that a pulse voltage of approximately 3.3 V, represented by the vertical dashed line in Fig. 3, is required to remove an individual adatom for the particular scanning parameters used in this experiment.

#### Threshold Voltage Vs. Applied Offset

**Figure 4:**
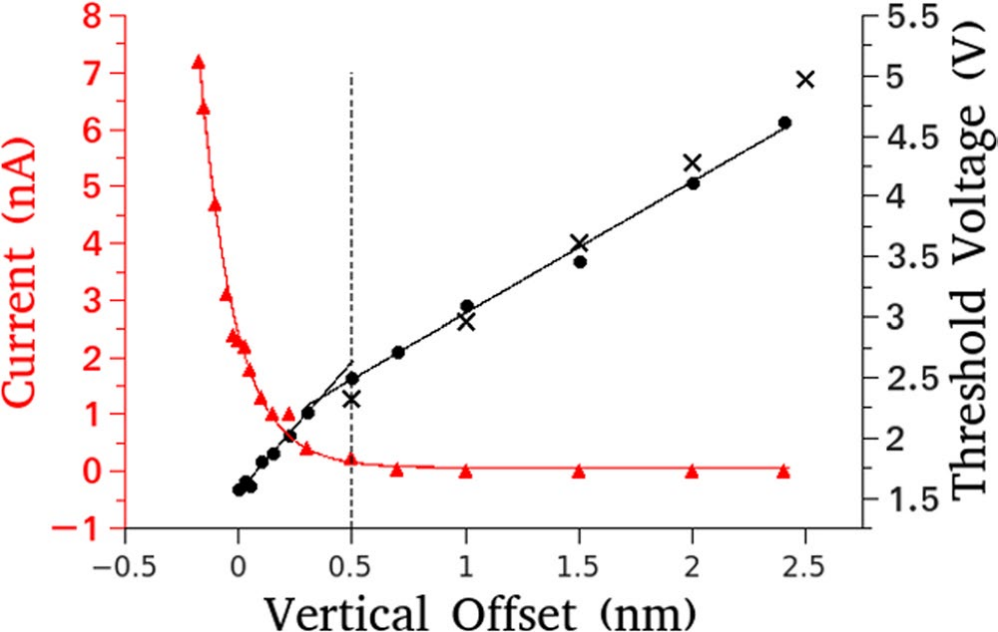
Red triangles: The dependence of tunneling current on applied offset indicates that at a tip offset of 0.5 nm is sufficient to remove the tip from the tunneling regime; Black Circles: Experimental relationship between the threshold voltage and tip offset is linear and divided into two regions by the dashed line which corresponds to the end of the tunneling regime; Black crosses: FEM simulations (*V* = 0.43 V) of the threshold voltage and vertical offset, using only an electric field, closely agree with the experimental relationship outside of the tunneling regime.

The red triangle in Fig. 4 demonstrates the dependence of the tunneling current on the applied offset, note the absence of a tunneling current after an offset of approximately 0.5 nm is applied. The dependence of the threshold voltage on the tip-surface distance has been explored. An offset has been applied to the tip and the pulsing voltage has been increased until adatom removal is observed. Subsequently, further offsets have been applied and once again the threshold voltage is found. The threshold voltage for the removal of oxygen adatoms increases as the distance between the surface and tip increases as shown in Fig. 4 by the black circles. This relationship has two distinct regions, reflected in the change of slope, indicated by the dashed vertical line at an offset of 0.5 nm. Below an offset of 0.5 nm the tunneling current contributes to adatom removal whereas at for an offset above 0.5 nm the tunneling current does not contribute to the removal of the oxygen adatoms. The linear relation between vertical offset and threshold voltage with no tunneling current is in agreement with that of the electric field mechanism theory, seen in Eq. (5), which predicts a linear relationship. In the absence of a tunneling current oxygen adatom removal is only observed at positive bias voltage (applied to the surface relative to the tip).

#### Determining the Potential barrier

**Figure 5:**
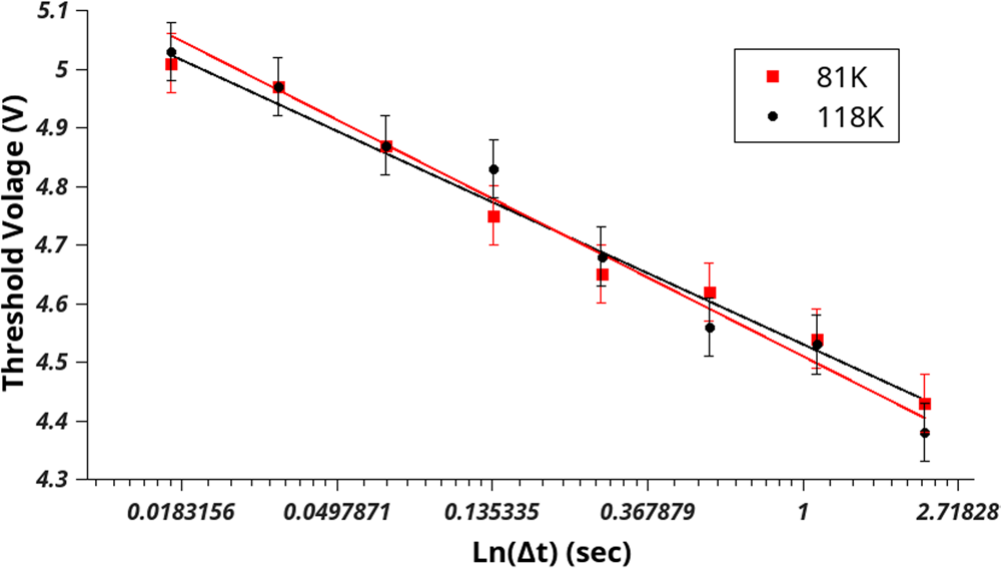
The dependence of threshold voltage on the duration of bias voltage pulse measured at two temperatures: red and black circles correspond to 81 K and 118 K respectively. The dependencies are fitted by linear functions.

To penetrate the surface, an oxygen adatom needs to overcome a potential barrier, *W*. The magnitude of this barrier can be modified by applying an electric field. If the probability of an oxygen adatom being removed follows the relationship in Eq. (2), measuring the threshold voltage as a function of the pulse length, Δt, will yield a linear relationship. Such an experiment has been performed at 121 K and 81 K and is depicted in Fig. 5. Turning to Eq. (4), the intercept of the *V* vs ln(Δt) graph contains two terms. The temperature dependent term provides only a small contribution, as the intercept only changes by ~0.1 V for the measurements at 121 K and 81 K, and hence, this term is omitted. Therefore, the intercept provides an approximate value for $W\cdot \frac{d+{Z}_{off}}{aq}$. Considering that the slope provides a value for ${k}_{b}T\cdot \frac{d+{Z}_{off}}{aq}$, an approximate value for *W* is be determined to be 0.45 eV.

### FEM Simulation Results

**Figure 6:**
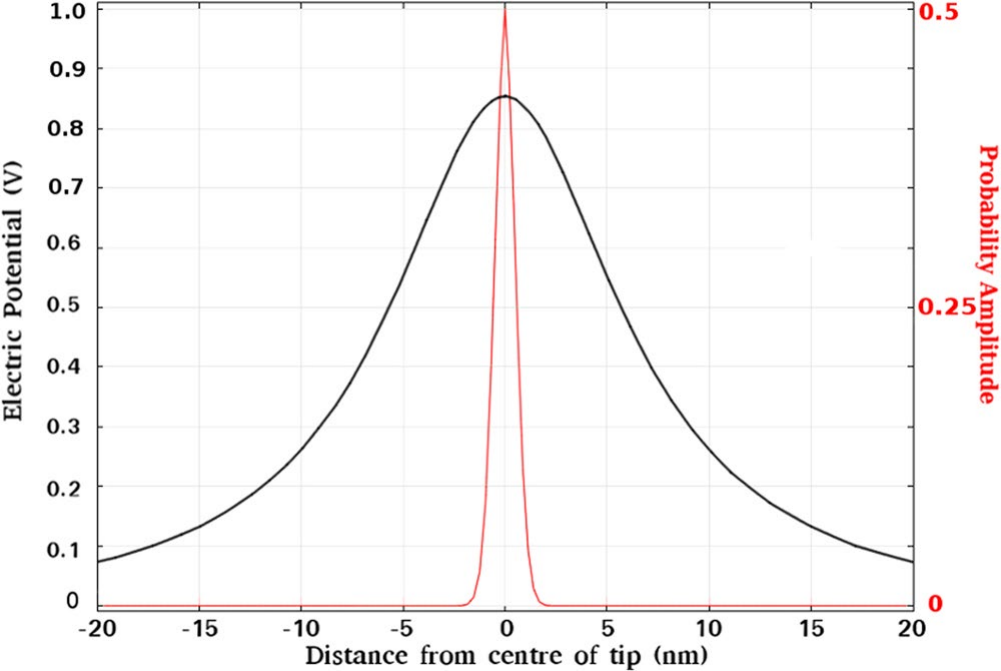
The electric potential curve (black) corresponding to the threshold voltage, is used to generate the probability amplitude shown in red using Eq. (2) where *W* is set to equal the peak value of the electric potential and the pre-exponential factors set so that the probability peak is a maximum at 0.5. This probability curve then closely represents the probability to remove an adatom at the threshold voltage. This curve supports the narrow spot diameter of ≈1 nm observed in experiment when adatoms are removed from the surface.

FEM simulations^35^ of the electric field generated between the tip and surface have been performed in order to shed light on the mechanism behind the removal of oxygen adatoms from the MoO_2+*x*_/Mo(110) surface. Figure 6 illustrates an example of the electric potential (black curve) calculated along a line, parallel to the surface, at the adatom height above the surface. The corresponding probability to remove an oxygen adatom from the surface (red curve) calculated using Eq. (2) is shown in Fig. 6. The pre-exponential factors are set such that the probability curve peak is normalised to 0.5, which coincides with the probability of removing one adatom at the threshold voltage. Although the spatial distribution of the potential curve demonstrates the broad peak located below the STM tip apex with a full width half maximum (FWHM) of approximately 15 nm, the FWHM of the probability curve is much narrower. The FWHM of the probability curve is approximately 1 nm. Considering the adatom directly below the tip apex has a probability of 0.5, i.e. threshold, the probability of its nearest neighbours, which are at most 0.5 nm away, being removed is at most 0.25. This serves to highlight that the electric field can give rise to extremely high-resolution removal of oxygen adatoms and is in good agreement with the resolution of individual oxygen adatoms removal observed in experiment, see Figs 1 and 2.

A reference threshold value of 3.3 V for an offset of 1 nm was taken from the threshold voltage vs. applied offset experiment, black circles in Fig. 4, to simulate the relationship between the spot size and the applied bias voltage. The spot size in which adatoms are removed is calculated as the area in which the applied electric potential exceeds the threshold electric potential in the plane of the adatom, parallel to the surface. An example of this calculation can be found in the methods section. The comparison between these computational results and the experimental results can be seen in Fig. 3. To simulate the relationship between the applied offset and the corresponding threshold voltage, the electric potential at the position of the adatom is calculated as a function of applied bias voltage, this is repeated for different tip offsets. Interpolating the data allows one to select a potential value and determine the bias voltage required to generate the potential in question at various tip-surface offsets. A potential value of 0.43 V best fits the experimental data. The black crosses in Fig. 4 depict the calculated linear relationship between the threshold voltage and the tip-surface distance, which is in good agreement with the experimental findings (black circles). The FEM results, obtained solely from applying an electric field, are in agreement with the experimental results, indicating that the process of oxygen adatom removal from the MoO_2+*x*_/Mo(110) surface can be realised without the influence of tunneling electrons.

## Discussion

Oxygen adatom removal is only observed when a positive bias is applied to the sample. Given that the oxygen adatoms are negatively charged and that the electric field acts perpendicular to the surface due to its conductive nature, the net force on the adatoms acts perpendicular and towards the surface. Large surface area scans before and after pulsing indicate that the density of adatoms in the regions surrounding the pulsed areas does not change upon pulsing outside of the tunneling regime, indicating there is no lateral diffusion in this regime. Pulsing at high voltage/current in the tunneling regime can lead to lateral diffusion, likely due to local heating of the surface. When the polarity of the pulse voltage is reversed, no adatoms are observed to be removed, when outside of the tunneling regime. Oxygen adatoms have been observed to transition to an intermediate state before being fully removed from the surface. This state occurs when pulsing close to the threshold voltage and results in a reduction in the apparent height of the oxygen adatom in STM images, indicating that either, the oxygen adatom is now within the surface layer or has changed its electronic state, likely due to chemical bonding.

The penetration of oxygen atoms through metal and metal oxide surfaces has received attention from a theoretical viewpoint^36,37^. The energy barrier for oxygen incorporation depends on the oxygen adatom distance to the underlying surface layer and the bond lengths of the atoms within the layer which the oxygen adatom will penetrate, and hence, the substrate electron density. A larger electron density results in a larger barrier. Several factors affect the electron density at the point where the adatom crosses into the substrate, such as the lattice constant, lattice structure and material. In transition metals and their oxides, a 10% increase in lattice constant reduces the barrier by almost 30%^37^. Surface relaxations thus play an important role in determining the substrate electron density. Several different crystal lattice structures have been studied^36,37^, the rocksalt structure is very homogeneous and close-packed, leading to high energy barriers. While in the rutile, bixbyite, and spinel structures there are “open channels”, which can be envisioned as vacancies, through which the oxygen can diffuse easily and thus have lower energy barriers for oxygen incorporation^37^. Introducing a defect in the third layer of NbO(111) reduces the energy barrier from 4 eV to 1.2 eV^37^. Furthermore, the presence of an oxide-overlayer can increase or decrease the barrier, depending on the induced modification of the metal lattice substrate. For example, in the case of Nb(110), the barrier increases from 1.2 eV to 7.4 eV after oxidation. Whereas the barrier for incorporation into the Cu and CuO_2_ lattices are 0.9 and 0.04 eV^36,37^, respectively.

**Figure 7:**
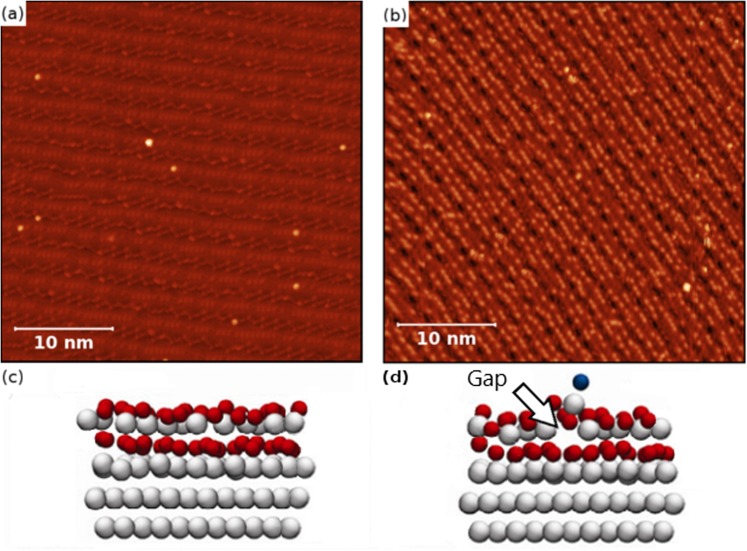
**(a)** STM image of the MoO_2_/Mo(110) surface displaying the bright and dark row features. **(b)** STM image of MoO_2+*x*_/Mo(110), the bright features on the bright rows correspond to oxygen adatoms. **(c)** DFT simulation of MoO_2_/Mo(110) surface **(d)** DFT simulation of MoO_2+*x*_/Mo(110) surface. Note the Mo (white) atom directly below the O adatom (blue) is shifted from its lattice position.

Pulsing of the MoO_2_ surface without any adatoms did not result in any oxygen atom transitioning below the surface. The relative ease (barrier calculated to be 0.45 eV) at which oxygen adatoms on the MoO_2_ surface can penetrate the surface is concluded to be due to how the adatoms modify the nearby MoO_2_ lattice. Density functional theory (DFT) calculations have previously been performed in order to understand the structure of the molybdenum dioxide layer^38^ and the over-oxidised MoO_2+*x*_ surface^27^. These calculations can shed light on the observation of the removal of oxygen adatoms via pulsing with the STM tip. The simulations, seen in Fig. 7(c,d), indicate a deformation of the lattice structure of the topmost molybdenum layer immediately around the oxygen adatom. The molybdenum atom below the oxygen adatom relaxes from its position in the molybdenum layer to the oxide layer above it, changing its oxygen coordination. This deformation results in the opening of a “channel” in the topmost molybdenum layer for the adatom to pass through. It is suggested that defected structures such as this, reduces the barrier for an adatom to penetrate into the underlying oxygen layer as the electron density is reduced in that region. Ion transfer in a crystal lattice under an applied electric field is characteristic of ion conductivity and electrodiffusion. Oxygen mobility through vacancies is the basis of oxide ion conductivity in numerous fluorite, perovskite and pyrochlore systems^39^. The activation energies for such ionic conductivity’s were found to be in the range of 0.7 to 1.6 eV^40^, which is of the same order of magnitude as the potential barrier height obtained in our experiments (0.45 eV).

The FEM model simulating the electric field effect yields convincing results despite its simplicity. Disparity between the FEM simulation and experimental results are suspected to be due to the differences in the simulation parameters such as the tip shape. Furthermore, the surface is neither perfectly flat or conducting, which would lead to a different potential energy being present in the vicinity of the surface. The value for the adatom-surface distance, *a*, in the simulations was intuitively taken as the distance between an oxygen adatom and the topmost molybdenum layer, this assumes the potential barrier is a maximum in the plane of the molybdenum layer, referred to as the crossing point. However, in a non-uniform system, such as this one where the oxygen adatom deforms the topmost molybdenum lattice structure, the crossing point may not lie in the same plane as the topmost molybdenum atoms. Variations of charge distribution on the atomic scale can affect the spatial distribution of the electrostatic field generated by STM tip which can influence the potential barrier. Nevertheless, the simulation is in good agreement with the experiment, suggesting that our model, which is based solely on the electric field, is correct.

Local modification of oxide compounds via oxygen diffusion induced by an external electric field has been previously observed in numerous experiments^41,42^ on resistive switching and filament formation in memristor structures. Memristor structures (metal-insulator-metal) are mostly based on doped oxide insulator films^31,32^. Memristor switching is usually initiated via oxygen vacancy diffusion induced by an electric field in an interface layer of 1–10 nm in thickness. In order to reveal the memristor like resistive switching of the MoO_2_ structure, we have performed experiments on a molybdenum single crystal with a silver point contact (see supplementary section). The MoO_2_ layer in this memristor experiment has oxygen vacancies which support the diffusion of oxygen ions through it. Resistive switching has been observed at bias voltages in the range of 0.5–1.5 V, which again supports the energy barrier previously obtained in our experiments.

## Conclusion

The oxygen adatoms on the MoO_2+*x*_/Mo(110) surface demonstrate high mobility. Pulsing the oxygen adatoms with the STM tip leads to the removal of the adatoms from the surface. This only occurs at positive sample bias and is observed in the absence of the tunneling current. The bias polarity dependence, the absence of lateral surface diffusion and the presence of an intermediate state indicates that the adatom penetrates the topmost Mo layer due to the electric field. FEM simulations of the electric field generated between tip and surface accurately describe the experimental results, indicating that the electric field is solely responsible for the observed removal of the oxygen adatom, which is pushed below the terminating layer. DFT calculations show that the oxygen adatom modifies the underlying Mo layer structure, which in turn opens up a “channel” in the Mo layer, which can reduce the energetic barrier for the adatom to transition to the oxygen layer below.

The atomic resolution capability of this method of oxygen adatom removal is due to the exponential dependence of the probability to transition on the applied bias voltage. The minimum spot diameter in which adatoms are removed from the surface is quite small, just 0.5 nm in diameter. The potential barrier for oxygen adatom incorporation into MoO_2+*x*_/Mo(110) was found to be approximately 0.45 eV. This localised mechanism allows for the implantation of atoms without strongly influencing the substrate.

## Methods and Theory

### Experimental Design

An atomically-clean single crystalline Mo(110) (Surface Preparation Laboratory) surface was prepared by *in-situ* annealing at 1275 K. The preparation chamber used in experiments has a base pressure of 5 × 10^−11^ mbar at 300 K. The sample was heated by electron beam bombardment and temperatures were measured using an optical pyrometer (Ircon UX20P, emissivity 0.35). The clean Mo(110) surface was verified by low-energy electron diffraction (LEED) and STM before oxidation. Subsequently, the sample was oxidised, using O2 gas with 99.998% purity, at 1250 K in an oxygen partial pressure of 1 × 10^−7^ mbar for 2 minutes and allowed to cool to room temperature, resulting in a clean MoO_2_/Mo(110) surface termination^43^. The MoO_2_ (010) surface, which is detailed in length by *Radican et al*.^43^ and depicted in Fig. 7(a), exhibits an O-Mo-O trilayer structure and forms well-ordered oxide nanorows which appear in STM images as bright rows with dark rows in between. These rows follow the^1^ direction of the Mo(110) substrate. In order to obtain oxygen adatoms on the MoO_2_/Mo(110) surface, the sample was then exposed to an oxygen environment of 1 × 10^−7^ mbar for 2 minutes at room temperature. This results in the formation of an oxygen-rich MoO_2+*x*_/Mo(110) surface exhibiting oxygen adatoms^27^ which is depicted in Fig. 7(b). Oxygen adatoms appear as small bright dots on the MoO_2+*x*_ surface and make up the vast majority of the observed features. They can be seen in greater detail in the images presented in Fig. 1. During the development of the oxidation procedure to obtain the MoO_2+*x*_/Mo(110) surface, the MoO_2_/Mo(110) surface was exposed to different doses of oxygen and there is a strong link between the number of these small bright dots and the dose of oxygen which the surface was exposed to. Further details correlating the small bright features to oxygen adatoms can be found in the supplementary section. Only these features, which can be easily distinguished from others, were the subject of the pulsing experiments.

The STM experiments were performed at liquid nitrogen temperature (78 K), using a commercial STM from Createc, in an ultra-high-vacuum (UHV) system with a base pressure of 2 × 10^−11^ mbar). An electrochemically etched single crystalline tungsten tip^44,45^ was utilized in the following pulsing experiments. The bias voltage is applied to the sample with respect to the tip. No drift corrections have been applied to any of the STM images presented in this work. Images were obtained in constant current mode. Further details of the preparation of the MoO_2+*x*_/Mo(110) surface and STM experimental conditions are outlined elsewhere^27^.

Pulsing involves applying a bias, for some period of time, to the surface whilst the tip is in its vicinity. Different bias voltage and tip-surface distance change the probability of removing oxygen atoms and the area from which the adatoms are removed. Prior to pulsing, the surface is scanned. The tip is then positioned directly above an oxygen adatom. In the tunneling regime the thermal influence of the tunneling current, inelastic electron scattering and the electric field generated between the STM tip and the surface contribute to the removal of oxygen adatoms. In order to isolate the contribution of the electric field, a vertical offset is applied to the STM tip to remove it from the tunneling regime for the duration of the applied voltage pulse. The vertical offset is not the absolute tip-surface distance but is the distance the tip is moved from its initial position in the scanning tunneling regime. In order to ensure consistency in this height, the initial scanning bias voltage and tunneling current were kept constant throughout all experiments (bias voltage = 1 V, tunneling current = 1 nA) and a tip offset of 1 nm (sufficient to remove the tip from tunneling regime) was used unless otherwise stated. The pulse duration for the following experiments was selected as 500 ms. Increasing the pulse duration above this value does not cause a significant change in the pulses’ effectiveness at removing an adatom^27^. Following pulsing, the surface is scanned again to determine if the adatom has been removed. The minimum voltage required to remove an adatom is referred to as the threshold voltage.

The stability of the tip position has been previously tested in our experiments^46,47^ on atom manipulation using the same microscope. The tip is approached into the tunneling regime and the feedback is then switched off. No changes in the tunneling current were detected during a 100 second interval. The error in applying a vertical offset is estimated to be no more than 0.1 Å. All experiments were performed 30 hours after filling the STM with liquid nitrogen, ensuring that the temperature is stable and therefore no thermal drift is present. The accuracy of the tip can be verified by scanning an area before and after pulsing and comparing the target position (the position selected on the first(before) STM image) and the real position (the position adatoms are removed from on the second(after) STM image). The target and real positions are within 1 Å, this high of precision can be observed in Fig. 1(c).

### Potential barrier

To penetrate the surface, an oxygen adatom needs to overcome a potential barrier, *W*. The probability of the oxygen adatom overcoming this barrier and penetrating the molybdenum surface layer to the oxide layer below depends on the frequency of attempts to overcome the barrier denoted by *f* (phonon frequency) and the time interval Δt in which attempts are made.

1 $$P=f\,{\rm{\Delta }}t\,A\,exp(-\frac{W}{{{\rm{k}}}_{{\rm{b}}}\,T})$$

*A* is the pre-exponential factor. The electric field generated by applying a negative bias to the tip with respect to the surface reduces this barrier. If the sample metal surface is taken to be a reference point at 0 potential, the potential barrier height reduction can be written as *W* − *qψ*, where *q* is the effective charge of an oxygen ion and *ψ* is the electric potential at the point of the oxygen ion. It follows that the probability of the oxygen adatom penetrating the surface in the presence of electric field *E* generated by the STM tip is:

2 $$P=f\,{\rm{\Delta }}t\,A\,exp(-\frac{W-{\rm{q}}\,\psi }{{{\rm{k}}}_{{\rm{b}}}\,T})$$

At the typical parameters of tunneling (tip apex is of the order of tens of nanometers according to Scanning Electron Microscope (SEM) experiments and the tip-surface distance, *d*, is approximately 1 nm) the electric potential can be be approximated by a simple model in which we consider the tip and surface as a parallel-plate capacitor. Then the potential at a distance of *a* from the plate is given by *ψ*:

3 $$\psi =V\,\frac{a}{d+{Z}_{off}}$$

*a* corresponds to the distance between the oxygen adatom and the metallic surface, taken to be the topmost Mo plane. *d* is the tunneling distance, while *Z*_*off*_ is any additional offset. Substituting for *ψ* in Eq. (2), taking the logarithm of both sides and solving for the *V* gives the following:

4 $$V=-\,\frac{(d+{Z}_{off})\cdot {{\rm{k}}}_{{\rm{b}}}T}{{\rm{q}}\,a}\cdot \,\mathrm{ln}({\rm{\Delta }}t)+\frac{(d+{Z}_{off})\cdot W}{{\rm{q}}\,a}+\frac{(d+{Z}_{off})\cdot {{\rm{k}}}_{{\rm{b}}}T}{{\rm{q}}\,a}\cdot \,\mathrm{ln}(\frac{P}{f\,A})$$

5 $$\therefore V\propto {Z}_{off}$$

indicating that an increase in applied offset causes the threshold voltage to increase linearly.

### FEM Simulations

FEM simulations^35^ of the electric field generated between the tip and surface have been performed in order to shed light on the mechanism behind the removal of oxygen adatoms from the MoO_2+*x*_/Mo(110) surface. In the FEM model, the tip is approximated as a conductive sphere and the MoO_2_ a perfectly flat metallic plane. An electric potential is applied to the tip and the electric field between tip and surface can then be determined. The electric potential at any point can be then calculated as a function of tip-surface distance, tip radius of curvature and bias voltage. This allows one to estimate the trends of the experimentally observed relationships, such as the threshold voltage for the removal of oxygen adatoms as a function of tip-surface distance. The electric potential is calculated at a distance of 0.4 nm above the first Mo layer of the O-Mo-O tri-layer according to DFT results^27^ seen in Fig. 7(d). The tunneling gap, the distance between the adatom on the tip, used in the simulation was 0.8 nm. In total a tip-surface distance of 1.2 nm is used prior to an offset being applied. This is then used as a zero point when calculating offsets in the simulations, for example, an offset of 1 nm corresponds to a total tip-surface distance of 2.2 nm. A tip diameter of 30 nm is used in the simulations. The tip diameter and tunneling gap were selected based on the simulations of spot size vs. applied bias voltage. Given that the oxygen adatom is negatively charged a negative tip bias (positive sample bias as in experiment) will force the adatom to move towards the surface. The tangential component of the force acting on the adatom is negligible due to the metallic conductivity of the surface, and hence, only the component of force perpendicular to the surface is considered. Using this principle, the electric field between the tip and the surface is simulated in order to get an estimate for the electric potential experienced by the oxygen adatom when a pulse is applied.

**Figure 8:**
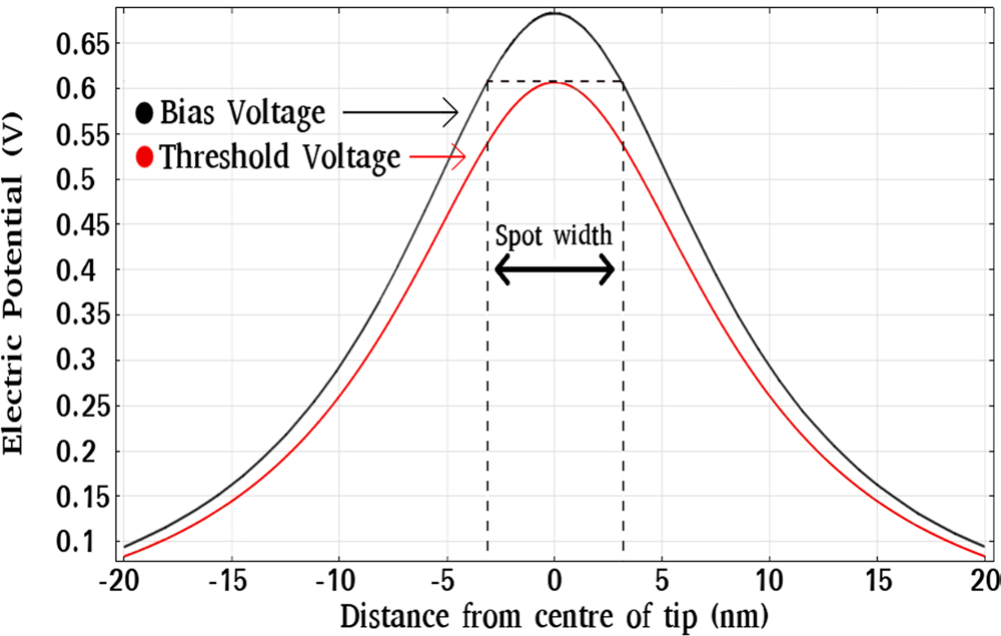
The spot diameter is determined by finding the diameter of the circular region in which the applied bias voltage (black) exceeds the threshold voltage (red).

In order to simulate the relationship between the spot diameter and the applied bias voltage, which used a vertical offset of 1 nm, an experimental reference point is required. A reference point of 1 nm offset for an applied bias voltage of 3.3 V was taken from the threshold voltage vs. applied offset experiment, black circles in Fig. 4. Using this offset and bias voltage the electric potential is calculated in the plane parallel to the surface, containing the point of the adatom’s position, which is 0.4 nm above the surface. Bias voltages exceeding this threshold voltage are then simulated and their electric potentials in the same plane are calculated. The spot size is determined by calculating the region in the plane for which the magnitude of the potential of the applied bias voltage exceeds the magnitude of the potential of the threshold voltage. An example of this is demonstrated in Fig. 8, where a line profile has been taken through the center of the plane. This spot width calculated here corresponds to the diameter of the spot in which adatoms are most probable to be removed.

To simulate the dependence of the threshold voltage on the tip-surface distance, the electric potential directly below the tip at the adatom height of 0.4 nm above the surface is calculated as a function of the applied bias voltage for various tip-surface offsets. These dependencies can be interpolated in order to select a potential and determine the bias voltage required to generate said potential at different tip offsets. Comparing the resulting data for a wide range of potential values to the experimental results not only allows us to find the best match to experiment but also to estimate the potential required for the adatom to transition below the surface.

## Supplementary information

Supplementary information

## Data Availability

The datasets generated during and/or analysed during the current study are available from the corresponding author on reasonable request.
