# Supplementary material for: Surface modification on MoO2+x/Mo(110) induced by a local electric potential
Source: Sci Rep. 2019 Apr 17;9:6216. doi: 10.1038/s41598-019-42536-9 (PMC6470205; doi:10.1038/s41598-019-42536-9)
Supplement: Supplementary file 1 — Supplementary information [file 41598_2019_42536_MOESM1_ESM.pdf]

Surface modification on MoO_{2+x}/Mo(110) induced by a local electric potential

Sergey I. Bozhko[1,2,+], Killian Walshe[2,*,+], Natalia Tulina[1], Brian Walls[2], Olaf Lübben[2], Barry E. Murphy[2], Vladimir Bozhko[1], and Igor V. Shvets[2]

[1]Institute of Solid State Physics, Russian Academy of Sciences, Chernogolovka, Moscow District 142432, Russia
[2]School of Physics and Centre for Research on Adaptive Nanostructures and Nanodevices (CRANN), Trinity College Dublin, Dublin 2, Ireland
[*]walsheki@tcd.ie
[+]these authors contributed equally to this work

ABSTRACT

Oxygen adatoms on the MoO_{2+x}/Mo(110) surface are observed to be removed when a sufficiently large bias is applied between the scanning tunneling microscope tip and the surface. Experimental observations, such as the bias polarity dependence of adatom removal and the observation of an intermediate state, indicate that the adatom penetrates the surface oxide layer. Through the comparison of finite element method simulations with various experimental relationships, the electric field is concluded to be the sole contributor to adatom penetration into the surface oxide layer. The energetic barrier to this process is estimated to be approximately 0.45 eV in magnitude. Furthermore, the resolution of this phenomena is on the atomic scale: individual adatoms can undergo surface penetration whilst their nearest neighbour adatoms, separated by 5Å, are unaffected. The mechanism reported here has the advantages of not strongly influencing the substrate and is exceptionally localised, which can be beneficial for the synthesis of single atom devices.

Supplemental Section - Memristor Experiment

Local modification of oxide compounds via oxygen diffusion induced by an external electric field has been previously observed in numerous experiments[1,2] on resistive switching and filament formation in memristor structures. Memristor structures (metal-insulator-metal) are mostly based on doped oxide insulator films[3,4]. Memristor switching is usually initiated via oxygen vacancy diffusion induced by an electric field in an interface layer of 1-10 nm in thickness. In order to reveal the memristor like resistive switching of the MoO_2 structure we have performed experiments on a molybdenum single crystal with a silver point contact. A MoO_2 single crystal has been grown using gas transport reactions from the gas phase. The memristor structure in Fig.(1) was realised as a point contact of a Ag needle labelled as 1 and defect layer, labelled 2, which is about 1-10nm in thickness on top of the MoO_2 sample surface, labelled 3. The I-V curve of the memristor structure reveals switching between high (700Ω) and low (68Ω) resistance states at positive bias polarity in an interval of 1.0 - 0.4 V. Switching back is realised at negative bias polarity in an interval of 0.4 - 1.3 V. The damaged layer, 2, at a MoO_2 surface, 1, is an insulator and its conductivity is determined by the mobility of oxygen ions which can be driven by applied electric field. Applying an electric field causes diffusion of oxygen ions in the damaged layer, the direction of oxygen diffusion being determined by the polarity of the bias voltage. The threshold voltage, when switching is realised, is determined by the diffusion barrier.

The MoO_2 layer in this memristor experiment has oxygen vacancies which supports the diffusion of oxygen ions through it. Resistive switching has been observed at bias voltages in the range of 0.5 - 1.5 V, which again support the energy barrier previously obtained in our experiments. In contrast to the phenomena of oxygen adatom removal from the surface of MoO_{2+x}, memristor switching is bipolar; the switching of the bias voltage polarity causes a change of direction of the oxygen vacancy diffusion, resulting in a switch of the structure back into the initial resistive state. For oxygen adatom penetrating the MoO_{2+x} surface oxide layer, the local stress of the crystal lattice is reduced resulting in the lowering of the potential barrier and the closure of the “channel” seen in the DFT simulated images of the main paper, consequently the oxygen adatom can not pass through the topmost oxide layer to the surface when the bias is reversed.

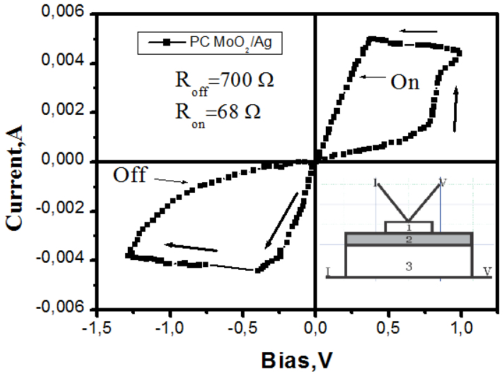

Figure 1

Supplemental Section - Oxygen Exposure Data

The exposure of the clean MoO_2/Mo(110) surface to an oxygen partial pressure of 1×10^{-7} mbar at room temperature lead to the formation of small bright features on the bright rows of the surface. The number of these features increased with the duration of the oxygen exposure. High purity oxygen (99.998%) was used in a system with a base pressure of 5×10^{-11} mbar to minimise contaminants. The MoO_2 surface is illustrated in Fig.(2a). In Fig.(2b) we present the surface after an initial exposure to oxygen for 40 seconds at a partial pressure of 1×10^{-7} mbar which results in a ~ 0.3 ML coverage. Fig.(2c) displays an increase in the number of bright features following a longer exposure to oxygen (80 seconds total) at the same partial pressure, the coverage is ~ 0.6 ML. Fig.(2d) displays the surface with a close to complete surface coverage which occurs after 2 minutes at the same partial pressure.

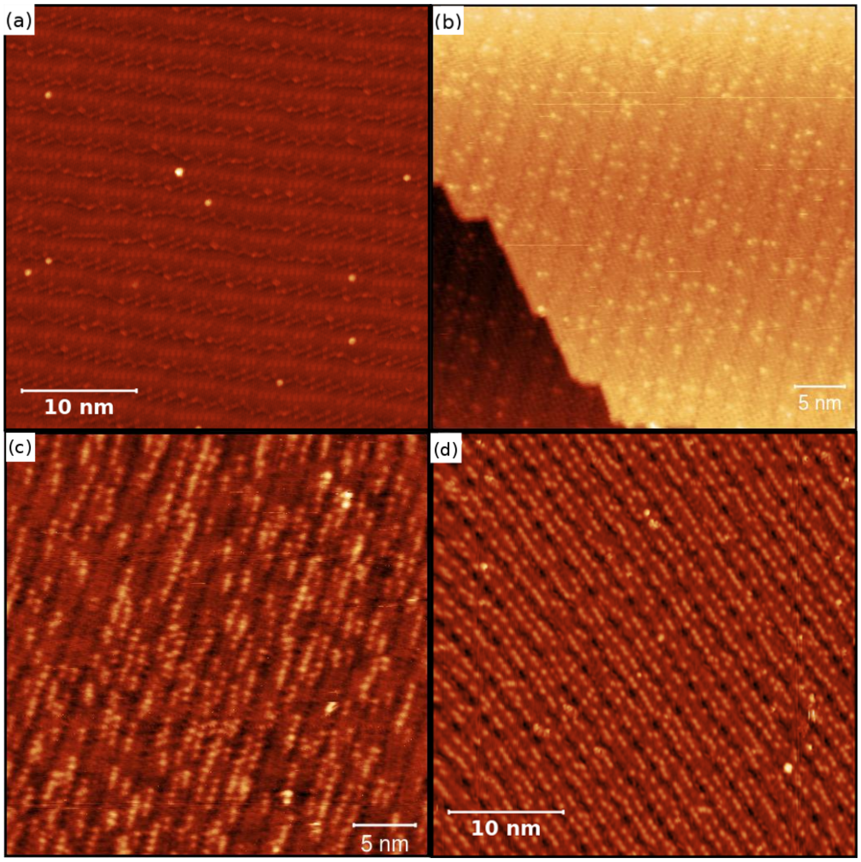

Figure 2

Altering the temperature that oxidation occurs influenced the resulting surface structure. Annealing the surface at 900 K for the duration of oxidation in an oxygen partial pressure of 1×10^{-7} mbar results in the formation of oxygen clusters forming in the grooves between the bright rows of the MoO_2/Mo(110) surface. These clusters were stable under pulsing experiments and can be observed in Fig.(3).

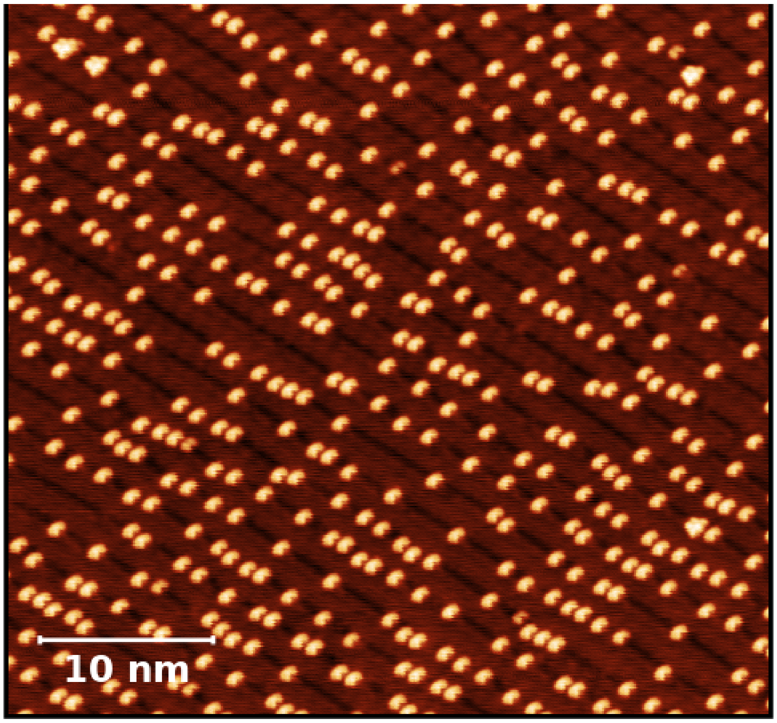

Figure 3

References

1. Nian, Y. B., Strozier, J., Wu, N. J., Chen, X. & Ignatiev, A. Evidence for an oxygen diffusion model for the electric pulse induced resistance change effect in transition-metal oxides. *Phys. Rev. Lett.* **98**, DOI: 10.1103/physrevlett.98.146403 (2007).

2. Yang, J. J. *et al.* Memristive switching mechanism for metal/oxide/metal nanodevices. *Nat. Nanotechnol.* **3**, 429–433, DOI: 10.1038/nnano.2008.160 (2008).

3. Tulina, N. *et al.* Static and dynamic effects of the resistive switchings in heterocontacts based on superconductive nd 2-x ce x CuO 4-y films. *Microelectron. Eng.* DOI: 10.1016/j.mee.2017.11.006 (2017).

4. Sawa, A. Resistive switching in transition metal oxides. *Mater. Today* **11**, 28–36, DOI: 10.1016/s1369-7021(08)70119-6 (2008).
